# Safety and efficacy of p62 DNA vaccine ELENAGEN in a first-in-human trial in patients with advanced solid tumors

**DOI:** 10.18632/oncotarget.16574

**Published:** 2017-03-25

**Authors:** Dmitry M. Ponomarenko, Irina D. Klimova, Yulia A. Chapygina, Viktoria V. Dvornichenko, Natalia V. Zhukova, Rashida V. Orlova, Georgy M. Manikhas, Alexandr V. Zyryanov, Lilya A. Burkhanova, Irina I. Badrtdinova, Basile N. Oshchepkov, Elena V. Filippova, Sergei V. Orlov, Sergei I. Kolesnikov, Albert A. Sufianov, Svetlana R. Baum, Olga Y. Zaitzeva, Andrey B. Komissarov, Mikhail P. Grudinin, Oleg I. Kiselev, Anatoly F. Tsyb, Franco Venanzi, Vita Shcherbinina, Andrey Chursov, Vladimir L. Gabai, Alexander M. Shneider

**Affiliations:** ^1^ Irkutsk State Medical Academy of Postgraduate Education, Irkutsk Regional Cancer Dispensary, Irkutsk, Russian Federation; ^2^ Saint-Petersburg City Clinical Oncology Dispensary, Saint-Petersburg, Russian Federation; ^3^ Saint-Petersburg University, Saint-Petersburg, Russian Federation; ^4^ Pavlov First Saint Petersburg State Medical University, Saint- Petersburg, Russian Federation; ^5^ Multidisciplinary Clinical Medical Center “Medical City”, Tyumen, Russian Federation; ^6^ Russian Academy of Sciences, Moscow, Russian Federation; ^7^ Federal Center of Neurosurgery, Tyumen, Russian Federation; ^8^ Sechenov First Moscow State Medical University, Moscow, Russian Federation; ^9^ Synergy Research Group, Moscow, Russian Federation; ^10^ Research Institute of Influenza, Saint-Petersburg, Russian Federation; ^11^ A. Tsyb Medical Radiological Research Center, Obninsk, Russian Federation; ^12^ Department of Biology MCA, University of Camerino, Italy; ^13^ CL Oncology, LLC, Moscow, Russian Federation; ^14^ CureLab Oncology, Inc, Dedham, MA, USA; ^15^ Department of Biochem, Boston University School of Medicine, Boston, MA, USA; ^16^ Department of Molecular Biology, Ariel University, Ariel, Israel

**Keywords:** cancer immunotherapy, chemotherapy, cancer vaccine, breast cancer, ovary cancer

## Abstract

Elenagen is a plasmid encoding p62/SQSTM1, the first DNA vaccine possessing two mutually complementing mechanisms of action: it elicits immune response against p62 and mitigates systemic chronic inflammation. Previously, Elenagen demonstrated anti-tumor efficacy and safety in rodent tumor models and spontaneous tumors in dogs. This multicenter I/IIa trial evaluated safety and clinical activity of Elenagen in patients with advanced solid tumors. Fifteen patients were treated with escalating doses of Elenagen (1- 5 mg per doses, 5 times weekly) and additional 12 patients received 1 mg dose. Ten patients with breast and ovary cancers that progressed after Elenagen were then treated with conventional chemotherapy. Adverse events (AE) were of Grade 1; no severe AE were observed. Cumulatively twelve patients (44%) with breast, ovary, lung, renal cancer and melanoma achieved stable disease for at least 8 wks, with 4 of them (15%) had tumor control for more than 24 wks, with a maximum of 32 wks. The patients with breast and ovary cancers achieved additional tumor stabilization for 12-28 wks when treated with chemotherapy following Elenagen treatment. Therefore, Elenagen demonstrated good safety profile and antitumor activity in advanced solid tumors. Especially encouraging is its ability to restore tumor sensitivity to chemotherapy.

## INTRODUCTION

Two mechanisms contributing to cancer have been extensively studied, inefficient anti-cancer immune response and chronic inflammation. Thus, two approaches to cancer therapy, stimulating anti-cancer immunity and eliminating chronic inflammation, are under active development. A novel biological agent, plasmid (DNA vaccine) encoding p62 (SQSTM1), acts through both of these mechanisms at the same time. Previously, it was demonstrated that anti-cancer agents may possess more than one activity, e.g. chemo-therapeutic agents inducing cancer cell death and modulating immune system at the same time.

Comparing to traditional (protein/peptide) vaccines, DNA-based vaccines have several advantages: they can induce both humoral and cellular immunity, they are safe, inexpensive and stable, and can be easily modified to enhance immune response [1–5]. There is already approved anti-melanoma vaccine for dogs (Oncept), and a number of ongoing phase I-II trials of DNA vaccines for various solid tumors in humans [2, 6]. However, being efficient in small animals, DNA vaccines often are not strong enough to control tumors in humans. Besides immunosuppressive tumor microenvironment, another apparent reason for vaccine inefficiency is that they exert selective pressure on cancer cells, which leads to the loss of the vaccine-encoded antigens (immune escape) and results in the tumor relapse [7, 8]. To avoid this problem, an effective antigen for a vaccine should be, besides evoking a robust immune response: 1) essential for cancer cells to avoid its loss through immunoediting; 2) dispensable for normal tissues to reduce the risk of toxicity, and 3) highly expressed in tumors as compared to the normal tissues [9].

We have chosen p62 (SQSTM1) protein as the antigen for a novel DNA vaccine (Elenagen). p62 is multifunctional protein which participates in selective autophagy and signal transduction, in particular, inflammatory response [10] [11]. p62 satisfies all the above requirements: 1) its levels are elevated in almost all human tumors tested so far; 2) tumors require p62 for growth and metastases, 3) p62 is dispensable for normal cells [3]. We have employed a plasmid DNA vaccine as a platform for p62 expression.

In our preclinical studies of Elenagen we found that: 1) it's intramuscular injection caused a strong anti-tumor effect in various animal models of breast cancer, melanoma, sarcoma and lung carcinoma [12]; 2) along with inhibition of growth of primary tumors, it also suppressed metastases [12], which are responsible for 90% of cancer-related deaths;3) importantly, beyond immune attack on tumors, Elenagen, unlike other vaccines, can also suppress chronic inflammation [13], a common feature of cancer that accelerates progression of the disease and worsens its outcome [14]; 4) apart from most of anti-cancer vaccines tested so far, it demonstrated efficiency not only in transplantable tumors in rodents, but also in spontaneous tumors of dogs that are very similar to humans [15].

Although DNA vaccines are generally considered safe enough [16], we performed broad-range preclinical safety studies of Elenagen in laboratory animals. The Elenagen administration was well-tolerated, without acute or chronic toxicity, allergic activity, and it did not cause embryonic toxicity and teratogenicity (Supplementary Table S1). There was no hematotoxic, hepatotoxic and nephrotoxic effects, as well as effects on carbohydrate and lipid metabolism. Furthermore, histological examination of brain, heart, lung, liver, kidney, spleen, thyroid and thymus did not find any anomalies. Overall, administration of Elenagen was not accompanied by any significant side effects (Supplementary Table S1).

Based on these efficiency and safety data in animals, we started the first clinical trial of Elenagen in patient with solid tumors.

## RESULTS

### Patients

The study included 27 patients: 15 patients in dose-escalation cohorts, and 12 in extension cohort; 2 patients have failed screening. Patient's characteristics are presented in Table 1. All patients had progressive metastatic solid tumors of one of 6 localizations: breast, colon, kidney, lung, skin (melanoma), and ovary. The most common tumors studied were breast (33.3%) and ovary (22.2%) cancers, and melanoma (22.2%). Patients enrolled had several (up to 8) chemotherapy schemes but were at the tumor progression state at the time of enrollment (Table 1).

**Table 1:** Patient characteristics

Patient characteristics	Patients (*n*=27)
**Gender** Female Male	21 (77.8%) 6 (22.2%)
**Age (yrs)** Medium Range	48 37-65
**Primary tumor** Breast Colon Kidney Lung Melanoma Ovary	9 (33.3%) 3 (11.1%) 1 (3.7%) 2 (7.4%) 6 (22.2%)
**Duration of disease (yrs)** Medium Range	3 1-13
**No. of prior lines of anticancer therapy** Medium Range	3 1-8
**Karnofsky Performance Status** Medium Range	80 70-100

### Safety

All patients included in the study completed at least one course (5 injections) of Elenagen vaccinations, and no discontinuation of the treatment occurred. No serious AEs caused by vaccination in dose range of 1-5 mg were observed in any of 27 patient of the study. There was no any acute toxicity or manifestations of cardiotoxicity. Autoimmunity (dsDNA antibody) was also within a normal range (not shown). The most common treatment-related grade 1 AE were injection site erythema or swelling, nausea, fatigue and fever; these events were independent on dose, transient, and resolved without treatment (Table 2). Thus, based on these data we concluded that Elenagen is safe and without dose-limited toxicity in the range of 1-5 mg per injection per week.

**Table 2:** Treatment-related adverse events

Toxicity	Grade 1	Grade 2	Grade 3	Grade 4
Injection site erythema or swelling	3 (11.1%)	0	0	0
Nausea	3 (11.1%)	0	0	0
Fatigue	3 (11.1%)	0	0	
Fever	3 (11.1%)	0	0	0
Patients with AE	9 (33.3%)	0	0	0

### Tumor response

#### Dose-escalation study

In the first part of the study, patients with different solid tumors were subjected to escalated doses of Elenagen and their tumor response was monitored by RESIST 1.1 criteria every 8 wks until tumor progression. The best overall response observed was SD (stable disease): it happened in 3 of 5 patients in 1^st^ cohort (1 mg), 1/5 patients in 2^nd^ cohort (2.5 mg), and 3/5 patients in 3^rd^ cohort (5 mg) (Table 3). The maximal duration of SD observed in the 1^st^ cohort was 32 wk, in the 2^nd^ cohort - 24 wks, and in 3^rd^ cohort - 8 wks. Although small size of the cohorts does not allow to make reliable conclusion about dose-response effect, there is no apparent dependence at given Elenagen doses which is common for DNA vaccines [17, 18].

**Table 3:** Effect of elenagen dose escalation on antitumor response

Patient #	Gender, age	Dose, mg	Tumor type and characteristics	Tumor response by RESIST 1.1
1	F, 46	1	Breast cancer, ER-; PR-; HER2-	**Stabilization 8 wks**
2	F, 37	1	Breast cancer, ER-; PR-; HER2- BRCA1+ mutation	Progression
3	M, 47	1	Lung cancer, EGFR mutation	**Stabilization 8 wks**
4	F, 45	1	Cancer of descending colon	Progression
5	F, 37	1	Breast cancer ER-; PR-; HER2-	**Stabilization 32 wks**
6	F, 64	2.5	Rectosigmoid cancer of the colon	Progression
7	F, 52	2.5	Breast cancer, ER-, PR-, HER2 +	Progression
8	F, 45	2.5	Ovary cancer	**Stabilization 24 wks**
9	M, 58	2.5	Rectosigmoid cancer of the colon, K-RAS/N-RAS mutation	Progression
10	M, 49	2.5	Lung cancer	Progression
11	F, 44	5	Breast cancer, ER-; PR-; HER2-	Progression
12	F, 51	5	Breast cancer, ER+;PR+; HER2-	Progression
13	F, 43	5	Breast cancer, ER-; PR-; HER2+	**Stabilization 8 wks**
14	F, 35	5	Breast cancer, ER-, PR-, HER2+	**Stabilization 8 wks**
15	F, 47	5	Ovary cancer	**Stabilization 8 wks**

Patient's tumor localization in the first 3 cohorts was selected randomly according to protocol inclusion/exclusion criteria (Supplementary Table S2) and patient's availability. SD was observed in different tumors: in 4 of 8 patients with breast cancer (PFS from 8 to 32 wks), in 2 of 2 patients with ovary cancer (PFS 8 and 24 wk), in 1 patients of 2 with lung cancer (8 wk); and in none of 3 with colon cancer (Table 3), demonstrating potentially broad anti-tumor activity of Elenagen. Furthermore, among major subtypes of breast cancer, SD was observed in 2 of 4 patients with triple-negative, in 1 of 2 with Her2 positive, and in 1 of 2 ER^+^PR^+^Her2^−^ tumors (Table 3), indicating that all major breast subtypes are sensitive to Elenagen.

#### Extended cohort study

Based on these pilot data on Elenagen efficiency, we extended our clinical study with 12 patients using the lowest efficient dose (1 mg). The patients selected were with ovary cancer, melanoma and neuroendocrine renal cancer (Table 3). The efficiency of Elenagen in all 27 patients (combined 1^st^ and 2^nd^ parts of the study) is presented in Table 4. Besides breast cancer, SD was also observed in 4 from 6 patients with ovary cancer, in 2 from 6 patients with melanoma, and 1 patient with neuroendocrine renal cancer; in total, 12 patients from 27 (44%) demonstrated SD for at least 8 wks. Remarkably, some patients with breast, ovary and renal cancer achieved tumor control for more prolonged period, from 24 to 32 wks (4 of 27 patients) thus reaching clinical benefit rate of 15%. Based on these data, we concluded that Elenagen as a single agent can evoke tumor control in a range of solid tumors: breast, ovary, renal, lung and melanoma, but without apparent activity in colon cancer.

**Table 4:** Efficacy results of treatment with elenagen only

Tumor localization	Tumor stabilization (8 wks)	Duration of stabilization, wks	
		Median	Max
Breast	4/9 (44.4%)	12	24
Ovary	4/6 (66.7%)	12	32
Melanoma	2/6 (33.3%)	8	8
Lung	1/2	NA	8
Renal (neuroendocrine)	1/1	NA	24
Colon	0/3	0	0
All	12/27 (44.4%)	NA	NA

NA – not applicable

### Effect of chemotherapy on tumor response following Elenagen treatment

Seven breast cancer patients and three ovarian cancer patients who progressed after one or several rounds of Elenagen treatment were administered conventional chemotherapy on compassionate basis. The rational for this was that immunotherapy can synergize with chemo- and radiotherapy *via* several mechanisms (see refs [19–21] for review and Discussion below). We found that patients with breast and ovarian cancers who failed several rounds of previous chemotherapy and were considered treatment-refractory, however, demonstrated rather prolonged tumor control (SD) (up to 28 wks) when treated with same chemotherapy regimen again (Table 5, 6). For example, 3 patients with triple-negative breast cancer (05-001, 005, 012) responded to conventional DC regimen (doxorubicin-cyclophosphamide) after failing it as first-line chemotherapy (Table 5). Accordingly, a patient with hormone-sensitive breast cancer (05-014) who stopped to respond to tamoxifen, restored sensitivity to it (Table 5). Ovarian cancer patients who failed platinum- and taxan-based treatments restored sensitivity to carboplatin (05-008, 016, Table 6). Some patients with breast and ovarian cancer also responded to new therapy regimens: eribulin in breast cancer and irinotecan for ovarian cancer (Table 5, 6). Therefore, even if Elenagen did not stop tumor progression as a monotherapy or when tumors progressed after multiple rounds of Elenagen treatment, the disease still could be controlled by chemotherapy even if it stopped to work before. This may indicate that Elenagen could become the first agent restoring tumor sensitivity to chemotherapy. So far, this can be applied to breast and ovary cancers and several anti-neoplastic drugs such as cyclophosphamide, adriamycin, platinum and taxan derivatives, but it may be a wider phenomenon.

**Table 5:** Effect of elenagen followed by chemotherapy in breast cancer

Patient	Age	Tumor markers	CA15-3 u/ml, basal	Prior therapy	Tumor response	
					Elenagen only	Chemotherapy
05-001	46	ER-; PR-; HER2-	3.5	RT, FDC, paclitaxel+ gemcitabine, cisplatin	SD 8 wks,	DC - SD 24 wk
05-002	37	ER-; PR-; HER2- BRCA1+ mutation	139	DC, RT, gemcitabine, cisplatin, avastin	PD	Eribulin - SD 12 wks; then cisplatin - SD 24 wk,
05-005	37	ER-; PR-; HER2-	19.5	FDC, docetaxel + cisplatin; gemcitabine +cisplatin; capecabin	SD 24 wks,	DC - SD 24 wks
05-012	44	ER-; PR-; HER2-	9.5	DC, paclitaxel	PD	DC - SD 12 wks, then eribulin+ trastuzumab SD - 20 wks
05-013	51	ER+, PR+, Her2-	694	RT, FDC, tamoxifen	PD	Anastarzol* - SD 28 wks
05-014	43	ER+; PR+; HER2-	33	FDC, RT, tamoxifen	SD 16 wks,	Tamoxifen - SD 20 wks*
05-015	35	ER-, PR-, Her2+	8	FDC, RT, carboplatin+herceptin	SD 8 wks,	Eribulin - SD 20 wks

SD – stable disease, PD – progressive disease; RT – radiation therapy, DC –doxorubicin+cyclophosphan; FDC – 5-fluorouracil+doxorubicin +cyclophosphan; * - still in the treatment (as of Dec 15^th^, 2016).

**Table 6:** Effect of elenagen followed by chemotherapy in ovary cancer

Patient	Age	Characteristics	CA-125u/ml, basal	Prior therapy	Tumor response	
					Elenagen only	Chemotherapy
05-008	45	Adenocarcinoma	593	Carboplatin, doxetacel, gemcitabine, avastin	SD, 24 wks,	Carboplatin - SD 16 wks; gemcitabine – 12 wks*
05-016	37	Endometroid adenocarcinoma	48	Cisplatin, avastin	SD, 8 wks	Carboblatin - SD 16 wks;*
05-018	66	Low- differentiated carcinoma	601	Cyclophosphamide, metotrexate, fluorouracil, cisplatin, taxol, oxaliplatin, doxorubicin	PD	Irinotecan - SD 12 wks; carboplatin – SD 12 wk*

SD – stable disease, PD – progressive disease; * - still in the treatment (as of Dec 15^th^, 2016).

## DISCUSSION

Among new methods to treat cancer, immunotherapy looks especially promising. Here we conducted a first-in-human trial of Elenagen, a DNA vaccine targeting p62. Although p62 is not oncogene per se under normal conditions, its overexpression is a common feature of various types of human tumors, and it correlates with tumor progression (see ref [3] for review). For example, in triple-negative breast and ovary cancers accumulation of p62 correlated with poor prognosis [22] [23]. It was also shown that upon oncogenic transformation p62 becomes necessary for tumor initiation and progression: Her2- -induced formation of breast cancer and RAS-induced formation of lung cancer are hampered in p62 knockout animals [24, 25]. Furthermore, fully transformed cells and tumors do not lose their dependence on p62, i.e. they become “addicted” to p62, a phenomenon known as non-oncogene addiction [26]. Accordingly, depletion of p62 suppressed growth of cancer cells *in vitro* and tumors *in vivo* in [27, 28].

Importantly, unlike tumor cells, p62 is dispensable for normal cells since p62 knockout animals are normally developed and demonstrate only minor anomalies (later-onset obesity) [29]. This indicates that, at least under normal conditions, other proteins can substitute some functions of p62 (e.g., p62 homolog NBR1 in autophagy) [30]. All these properties make p62 an excellent target for anticancer drug development. However, to our knowledge, there is only one study on chemically targeting p62 using XRK3F2 compound for treatment of myeloma [31]. The problem with targeting p62 by a drug is that it is multi-domain protein having at least 8 putative domains, and their roles in cancer are far from clear. In case of myeloma it is ZZ domain targeted by XRK3F2 appear to be important for their growth [31].

We have created a p62-encoding DNA vaccine. We found that p62 vaccine can generate immune response to p62 antigen [12], and anti-tumor response requires intact immune system since it lack in immunodeficient SCID mice (in preparation). Accordingly, in our study in dogs with spontaneous breast cancer we observed accumulation of T-lymphocytes within tumors [15]. We found that p62 vaccine has a broad range of anticancer activity in preclinical models of breast, lung carcinoma, melanoma and sarcoma [12]. Importantly, we also demonstrated p62 activity in spontaneous breast tumor in dogs [15]. Besides suppressing growth of primary tumors, Elenagen was able to markedly decreased development of metastasis [12].

We serendipitously uncovered another property of p62 plasmid - its ability to suppress chronic inflammation. We found that in a model of ovariectomy-induced osteoporosis in mice p62 plasmid injection prevented generation of pro-inflammatory cytokines and suppressed osteoporosis [13]; similarly, p62 alleviated inflammation and obesity caused by high-fat diets in rats (in preparation). It is well-known that chronic inflammation can play immune-suppressive role [32, 33] [34]. Furthermore, similar to p62 levels, elevated levels of inflammatory markers associated with worse survival, e.g., in breast [35] [36] and ovarian cancer [37] [38]. We hypothesize that, besides evoking immune response to a cancer antigen (like other vaccines), Elenagen can also alleviate chronic inflammation within a tumor thus enhancing the response. As a result of such dual action, p62 vaccine can be more potent than traditional vaccines.

The main goal of the present clinical trial was to assess Elenagen safety. Here we report that Elenagen demonstrated good safety profile (Table 2). Although patients selected for the phase I safety study were so called “terminal patients”, Elenagen still showed efficiency in breast cancer, having effect in all 3 subtypes of it (Table 4). Also, it was effective in ovary, lung, renal cancer and melanoma (Table 4). Applied as a monotherapy, Elenagen cased stabilization of disease for up to 32 wks in some patients with breast, ovary and renal cancer, although in most patients tumor stabilization was lasted for only 8 wks.

If we compare the results of this trial with other recent phase I/II trials of other immune-therapeutics (Supplementary Table S3), we can make several conclusions. First, Elenagen, similar to antibodies and other vaccines, and anti-CTLA4, in contrast to conventional therapeutics, does not evoke objective response (i.e. complete + partial response), but produces tumor control better than most other vaccines, antibodies, or anti-CTLA4 (Supplementary Table S3). Second, anti-PD1/PDL1 antibodies are more effective than other immune-therapeutics, but they however, have more severe side effects (Supplementary Table S3). But regarding specifically breast and ovarian cancers, Elenagen efficacy in tumor control is compatible with anti-PD1/anti-PDL1, but have much better safety (Supplementary Table S3).

During the trial we observed a phenomenon which, to our knowledge, has not been described in clinics before. When patients with breast and ovarian cancer were pretreated with Elenagen, they restored, at least partially, their sensitivity to conventional chemotherapy which they previously failed (Table 5, 6). There are several possible explanations for such effect. First, chemo-radiotherapy (e.g., doxorubicin) can enhance anti-p62 immune response by killing tumor cells *via* immunogenic cell death [21, 39]. Second, it is currently becoming evident that conventional chemo-radiotherapy, besides killing tumor cells directly, can also modulate immune response. For instance, cyclophosphamide, 5-fluorouracil, cisplatin can target immunosuppressive regulatory T and myeloid-derived cells, just enhancing immune response. Preconditioning vaccine and CAR-T therapies with these “chemotherapeutic” agents is already in clinical use [19–21]. Furthermore, some drugs such as gemcitabine and taxanes can directly stimulate immune system [39]. For instance, a vaccine directed to Her2-expressing mouse mammary tumors was potentiated by doxorubicin and paclitaxel [40]. Accordingly, treatment with doxorubicin after vaccination increased immune response to Her2 vaccine in clinics [41], and in patients with other tumors, chemotherapy applied after vaccination was more effective than vaccines or chemotherapy alone [42, 43].

Although Elenagen alone demonstrated anti-tumor activity in a range of solid tumors, in future trials we consider its combination with chemo-radiotherapy. We also believe that Elenagen should be synergistic with immune-checkpoint inhibitors. Combining immune checkpoint blockers (ICB) with vaccines targeting specific cancer antigens is an emerging approach [44]. So far, cancer vaccines are mostly failed in clinical trials because immune response they generate was apparently too weak to control tumor growth in patients owing to immuno-suppressive tumor microenvironment (e.g., due to activation of immune checkpoints). If so, one may expect that removing immune checkpoint blockade by ICB would enhance vaccine efficiency. Indeed, in preclinical models of cancer ICB significantly enhanced vaccine efficiency [45] [46]. Partially because of these findings, cancer vaccine field demonstrates resurgence [47], and there are currently several clinical trial of vaccine-ICB combination, e.g. with Prostvac, GVAX, CEA/MUC etc [44]. We believe that p62-encoding DNA vaccine Elenagen is a promising candidate within this booming area of research.

## PATIENTS AND METHODS

### Ethics

The clinical trial followed Declaration of Helsinki and Good Clinical Practice Guidelines. It was approved by Ethics Committee and registered by Russian Ministry of Health on 09.09.2014 (Clinical trial #506, Protocol E001).

It was also approved by local ethic committee of each participating institutions and written informed consent for participation in the study was obtained from all participants.

### Patients and study design

This multi-center, non-randomized, open-label, phase I/IIa trial was conducted in cohorts of patients with advanced metastatic solid tumors whose diagnosis was confirmed histologically. Inclusion/exclusion criteria for the patients in the study are presented in Supplementary Table S2. Only patients with radiologically measurable diseases after exhaustion of standard treatment options and in progression were enrolled into the study. The study consisted of two parts. The first part was dose-escalation study: 3 cohorts of patients with 5 patients in each received doses of 1, 2.5 and 5 mg, respectively. The second part was the expansion study containing 12 patients each receiving 1 mg dose. Each patient has received 5 i.m. injections of Elenagen (at concentration of 2.5 mg/ml in saline) once a week and then followed for 3 weeks before assessment of disease. If a patient demonstrated lack of disease progression, Elenagen injections were continued until tumor/metastases relapse. Standard regimens of chemotherapy were administered to 10 patients with breast or ovarian cancers, who either did not respond to Elenagen or demonstrated disease progression after a stabilization period on Elenagen treatment (s).

### Safety assessment

Screening and baseline assessments included evaluation of demographic data, medical and history, prior medications, a complete physical examination, vital signs, Karnofsky performance status, ECG, and clinical laboratory tests (including urinalysis and a serum pregnancy test if applicable), tumor history, primary diagnosis, and previous treatments. Safety assessments were performed after each injection. Adverse events (AE) severity was assessed according to the Common Terminology Criteria for Adverse Events (NCI-CTCAE), version 3.0. Safety assessments consisted of monitoring and recording all AEs; documenting concomitant medications; regular monitoring of hematology, blood chemistry, and urine values; periodic measurement of vital signs, Karnofsky performance status, ECGs; and performance of physical examinations. An ECG was recorded for each patient at screening, then 1 and 3 days after each dosing, and at the final visit. AE screenings and laboratory tests were performed weekly or as clinically indicated. Additionally, to assess inflammation and auto-immune response, CRP levels and antibody to dsDNA were measured, respectively, every week.

### Tumor response

Tumor measurements were assessed by computed tomography (CT) and evaluated at the centers using Response Evaluation Criteria in Solid Tumors (RECIST), version 1.1. Assessments were conducted at baseline and then 8 weeks after the start of the treatments; in case of tumor stabilization, monitoring was continued every 8 weeks (5 weekly injections followed by 3 weeks observation treatment) till tumor progression. Tumor response was defined as complete response (CR), partial response (PR), stable disease (SD), or disease progression (PD). SD was defined as to be maintained for 8 weeks and durable SD for more than 24 weeks. Clinical benefit was defined as CR, PR, and durable SD for more than 24 weeks.

## SUPPLEMENTARY MATERIALS TABLES


