# Supplementary material for: Safety and efficacy of p62 DNA vaccine ELENAGEN in a first-in-human trial in patients with advanced solid tumors
Source: Oncotarget. 2017 Mar 25;8(32):53730–9. doi: 10.18632/oncotarget.16574 (PMC5581145; doi:10.18632/oncotarget.16574)
Supplement: Supplementary file 1 [file oncotarget-08-53730-s001.docx]

Safety and efficacy of p62 DNA vaccine ELENAGEN in a first-in-human trial in patients with advanced solid tumors

**Supplementary Material**

**Table S1 . Preclinical study of toxicity of “Elenagen”**

| **Study** | **Animals/doses** | **Conclusion** |
| --- | --- | --- |
| Acute i.m. and i.p. toxicity | Rats, mice, guinea pigs 1, 5, 10, 50 ETD | No acute toxicity observed |
| Chronic toxicity upon i.m. daily administration for 90 days | Rats - 1,5,10, 50 ETD  Dogs – 1, 10 ETD | Low hazard  Low toxicity |
| Allergic activity | Guinea pigs, 1, 10 ETD | No anaphylactic shock or local allergic reaction |
| Immunological safety | Mice, 1,10 ETD, 5 times i.m | No effect on B- and T-cell response |
| Embryotoxicity and teratogenicity | Rats, 1, 10 ETD, 5-times i.m. | No embryotoxicity or teratogenicity |

*ETD – equivalent therapeutic doses*

**Table S2**. **Main inclusion/exclusion criteria**

| **Inclusion criteria** | **Exclusion criteria** |
| --- | --- |
| 1. Bladder, breast, cervix, colon, lung, ovary, pancreas, prostate, stomach cancers and melanoma. 2. Advanced or metastatic stages of disease lacking effective treatment 3. Applicability of RESIST 1.1 criterion to measure lesions. 4. Karnofsky perfomance status > 50 5. Life expectancy > 3 mo 6. Adequate bone marrow, heart, renal and liver functions 7. Chemo- and radiotherapy completion > 4 wks before enrollment | 1. Severe forms of allergic and autoimmune reactions in anamnesis 2. Acute infection during screening 3. Presence of another neoplasia 4. Brain metastases 5. Severe chronic diseases (i.e. cardiovascular, respiratory, liver etc) 6. Therapy by immune-biologics including cytokines, interferons etc during last 3 mo |

**Table S3. Comparison of Elenagen with other treatments in phase I/II studies**

| **Treatment** | **Tumor type** | | **Responses** | | | | | | | | | **Adverse Effects** | | **Refs** |
| --- | --- | --- | --- | --- | --- | --- | --- | --- | --- | --- | --- | --- | --- | --- |
|  |  |  | **Objective response** | | | **SD (wks)** | | **Tumor control, 24 wks** | | | |  |  |  |
| **Antibodies and Vaccines** | | | | | | | | | | | | | | |
| **Elenagen** | All Solid tumors (n=27) | | | 0 | | 44% (8 wks) | | | **15%** | | Grade 1 | | This study | |
|  | Breast+Ovary  (n=15) | | | 0 | | 53% (8 wks) | | | **20%** | | Grade 1 | |  |  |
| Anti-Semaphorin Ab (Vaccinex) | Solid tumors (n=42) | | | 0.5% | | 45% (8 wks); 19% (16 wks) | | | **7%** | | Grade 1-2 | | [[1](#_ENREF_1)] | |
| CD401 vaccine NY-ESO 1 | Solid tumors (n=45) | | | 0 | | 29% (4 wks) | | | **15.5%** | | Grade 1-2 | | [[2](#_ENREF_2)] | |
| GI-6207 CEA yeast vaccine | Solid tumors (n=25) | | | 0 | | 17% (16 wk) | | | **12.5%** | | Grade 1-2 | | [[3](#_ENREF_3)] | |
| TS poli-epitope vaccine | Solid tumors (n=21) | | | 5% | | 40% (12 wks) | | | **24%** | | Grade 1-2 | | [[4](#_ENREF_4)] | |
| PANVAC | Ovary cancer (n=14) | | | 0 | | 21% (12 wks) | | | **7%** | | Grade 2 - 85% | | [[5](#_ENREF_5)] | |
| **Checkpoints inhibitor blockers** | | | | | | | | | | | | | | |
| Anti-CTLA4 + radiation | | Prostate  (n=20) | | 4% | 21% (12 wks) | | **<18%** | | | Grade 3-4 -32% | | | [[6](#_ENREF_6)] | |
| Anti-CTLA4 + exemestane | | Breast Cancer (ER+) (n=26) | | 0 | 42% (12 wks) | | NA | | | Grade 3 - 27% | | | [[7](#_ENREF_7)] | |
| Anti-PD1 | | Breast cancer, triple negative (n=32) | | 18.5% | 26% (8 wk) | | **26%** | | | Grade 3 – 16%, 1 death | | | [[8](#_ENREF_8)] | |
|  |  | Ovary cancer  (n=20) | | 15% | 30% (24 wks | | **45%** | | | >Grade 3 – 40% | | | [[9](#_ENREF_9)] | |
| Anti-PDL1 | | Solid tumors  (n=135) | | 12% | 16% | | **28%** | | | >Grade 3-4 - 9% | | | [[10](#_ENREF_10)] | |
|  |  | Melanoma (n=52) | | 17% | 10% (24 wk) | | **27%** | | | NA | | |  |  |
|  |  | NSCL (n=49) | | 10% | 12% (24 wk) | | **22%** | | | NA | | |  |  |
|  |  | Ovary (n=17) | | 6% | 18% (24 wk) | | **24%** | | | NA | | |  |  |
|  |  | Renal (n=17) | | 12 | 41% (24 wk) | | **55%** | | | NA | | |  |  |
| Anti-PDL1+anti-CTLA4 | | NSCLC (n=102) | | 23% | 12% (24 wk) | | **35%** | | | Grade 3 - 36%, 3% death; 28% - treatment discontinuation | | | [[11](#_ENREF_11)] | |

**REFERENCES**

1. Patnaik A, Weiss GJ, Leonard JE, Rasco DW, Sachdev JC, Fisher TL, Winter LA, Reilly C, Parker RB, Mutz D, Blaydorn L, Tolcher AW, Zauderer M, et al. Safety, Pharmacokinetics, and Pharmacodynamics of a Humanized Anti-Semaphorin 4D Antibody, in a First-In-Human Study of Patients with Advanced Solid Tumors. Clinical Cancer Research. 2016; 22: 827-36. doi: 10.1158/1078-0432.ccr-15-0431.

2. Dhodapkar MV, Sznol M, Zhao B, Wang D, Carvajal RD, Keohan ML, Chuang E, Sanborn RE, Lutzky J, Powderly J, Kluger H, Tejwani S, Green J, et al. Induction of Antigen-Specific Immunity with a Vaccine Targeting NY-ESO-1 to the Dendritic Cell Receptor DEC-205. Science Translational Medicine. 2014; 6: 232ra51-ra51. doi: 10.1126/scitranslmed.3008068.

3. Bilusic M, Heery CR, Arlen PM, Rauckhorst M, Apelian D, Tsang KY, Tucker JA, Jochems C, Schlom J, Gulley JL, Madan RA. Phase I trial of a recombinant yeast-CEA vaccine (GI-6207) in adults with metastatic CEA-expressing carcinoma. Cancer Immunology, Immunotherapy. 2014; 63: 225-34. doi: 10.1007/s00262-013-1505-8.

4. Cusi MG, Botta C, Pastina P, Rossetti MG, Dreassi E, Guidelli GM, Fioravanti A, Martino EC, Gandolfo C, Pagliuchi M, Basile A, Carbone SF, Ricci V, et al. Phase I trial of thymidylate synthase poly-epitope peptide (TSPP) vaccine in advanced cancer patients. Cancer Immunology, Immunotherapy. 2015; 64: 1159-73. doi: 10.1007/s00262-015-1711-7.

5. Mohebtash M, Tsang KY, Madan RA, Huen NY, Poole DJ, Jochems C, Jones J, Ferrara T, Heery CR, Arlen PM, Steinberg SM, Pazdur M, Rauckhorst M, et al. A Pilot Study of MUC-1/CEA/TRICOM Poxviral-Based Vaccine in Patients with Metastatic Breast and Ovarian Cancer. Clinical Cancer Research. 2011; 17: 7164-73. doi: 10.1158/1078-0432.ccr-11-0649.

6. Slovin SF, Higano CS, Hamid O, Tejwani S, Harzstark A, Alumkal JJ, Scher HI, Chin K, Gagnier P, McHenry MB, Beer TM. Ipilimumab alone or in combination with radiotherapy in metastatic castration-resistant prostate cancer: results from an open-label, multicenter phase I/II study. Annals of Oncology. 2013; 24: 1813-21. doi: 10.1093/annonc/mdt107.

7. Vonderheide RH, LoRusso PM, Khalil M, Gartner EM, Khaira D, Soulieres D, Dorazio P, Trosko JA, Rüter J, Mariani GL, Usari T, Domchek SM. Tremelimumab in Combination with Exemestane in Patients with Advanced Breast Cancer and Treatment-Associated Modulation of Inducible Costimulator Expression on Patient T Cells. Clinical Cancer Research. 2010; 16: 3485-94. doi: 10.1158/1078-0432.ccr-10-0505.

8. Nanda R, Chow LQM, Dees EC, Berger R, Gupta S, Geva R, Pusztai L, Pathiraja K, Aktan G, Cheng JD, Karantza V, Buisseret L. Pembrolizumab in Patients With Advanced Triple-Negative Breast Cancer: Phase Ib KEYNOTE-012 Study. Journal of Clinical Oncology. 2016; 34: 2460-7. doi: doi:10.1200/JCO.2015.64.8931.

9. Hamanishi J, Mandai M, Ikeda T, Minami M, Kawaguchi A, Murayama T, Kanai M, Mori Y, Matsumoto S, Chikuma S, Matsumura N, Abiko K, Baba T, et al. Safety and Antitumor Activity of Anti–PD-1 Antibody, Nivolumab, in Patients With Platinum-Resistant Ovarian Cancer. Journal of Clinical Oncology. 2015; 33: 4015-22. doi: doi:10.1200/JCO.2015.62.3397.

10. Brahmer JR, Tykodi SS, Chow LQM, Hwu WJ, Topalian SL, Hwu P, Drake CG, Camacho LH, Kauh J, Odunsi K, Pitot HC, Hamid O, Bhatia S, et al. Safety and Activity of Anti–PD-L1 Antibody in Patients with Advanced Cancer. New England Journal of Medicine. 2012; 366: 2455-65. doi: doi:10.1056/NEJMoa1200694.

11. Antonia S, Goldberg SB, Balmanoukian A, Chaft JE, Sanborn RE, Gupta A, Narwal R, Steele K, Gu Y, Karakunnel JJ, Rizvi NA. Safety and antitumour activity of durvalumab plus tremelimumab in non-small cell lung cancer: a multicentre, phase 1b study. The Lancet Oncology. 2016; 17: 299-308. doi: 10.1016/s1470-2045(15)00544-6.
